# Comparison of the simplified International Index of Erectile Function (IIEF-5) in patients of erectile dysfunction with different pathophysiologies

**DOI:** 10.1186/1471-2490-14-52

**Published:** 2014-07-05

**Authors:** Zhengyan Tang, Dongjie Li, Xiaobo Zhang, Lu Yi, Xiangsheng Zhu, Xiangyang Zeng, Yuxin Tang

**Affiliations:** 1 Department of Urology, Xiangya Hospital of Central South University, Changsha, Hunan 410013, China; 2 Department of Geriatric Urology, Xiangya Hospital of Central South University, Changsha, Hunan 410013, China; 3 Department of Urology, The Second Xiangya Hospital of Central South University, Changsha, Hunan, China; 4 Department of The Andrology, Central Hospital of Xiangtan, Xiangtan, Hunan, China; 5 Department of Urology, The First People’s Hospital of Chenzhou, Chenzhou, Hunan, China; 6 Department of Urology, The Third Xiangya Hospital of Central South University, Changsha, Hunan, China

**Keywords:** Erectile Dysfunction, Pathophysiology, Psychogenic, Organic, IIEF-5

## Abstract

**Background:**

The simplified International Index of Erectile Function (IIEF-5) is a convenient, reliable and validated diagnostic tool for erectile dysfunction (ED). However, few studies focused on IIEF-5 in ED patients with different pathophysiological causes. ,We aim to compare the IIEF-5 score among ED patients with specific pathophysiologies in this study.

**Methods:**

The IIEF-5 score of 3,327 ED patients (median age 39 years) was analyzed. The primary causes of ED were determined by comprehensive diagnostic procedures in the urology/andrology clinics in five training hospitals. Patients with uncertain pathophysiologic cause were excluded.

**Results:**

176 patients were excluded, 3151 patients with ED history between 0.5 year and 20 years, were enrolled. The causes of ED was classified as psychogenic (59.2%), vasoculogenic (21.3%), neurogenic (4.1%), anatomical/structural (2.8%), hormonal (7.1%) or drug-induced (5.5%). A significant difference was detected in the median IIEF-5 score between psychogenic ED and organic ED (15 (IQR 13, 17) versus 12 (IQR 9.5, 14.5), P < 0.001). There was no significant difference of IIEF-5 scores among the organic groups (P = 0.073), or between arteriogenic and venogenic groups (13 (IQR 10.5, 15.5) versus 13 (IQR 11–15), P = 0.912 (adjusted α = 0.017)). However, the median IIEF-5 score of those with a mixed vascular cause was the lowest among vasculogenic patients (11 (IQR 8.5-13.5), scores for the three groups: P = 0.003.).

**Conclusions:**

The IIEF-5 scores of men with psychological ED are higher than those with organic causes, but there is no difference among patients with different organic pathophysiologies. Our data indicate that IIEF-5 is not a definitive diagnostic tool to discriminate the pathophysiological causes of ED.

## Background

Erectile dysfunction (ED) is defined as the inability to achieve and maintain an erection sufficient to permit satisfactory sexual intercourse [1,2]. A high prevalence of ED has been reported in many studies, and the situation could be even worse in the coming years, particularly in developing countries [3,4]. Although the release of phosphodiesterase type 5 inhibitors (PDE5-i) highly improved the treatment of ED patients with almost all kinds of pathophysiological causes, the high discontinuation rate of PDE5 inhibitors make it still essential to seek the causes for disease diagnosis and treatment [2,5-7].

The pathophysiology of ED includes vasculogenic, neurogenic, hormonal, anatomical, drug-induced and psychogenic causes in nature [8]. Identifying the pathophysiology of ED can significantly help to assess the modifiable risk factors and other medical conditions [9,10]. Furthermore, assessing the causes of ED is essential to cure patients with some certain types of ED [11].

The International Index of Erectile Function (IIEF) and the simplified International Index of Erectile Function (IIEF-5) are widely used, validated, self-administered questionnaires, and havebeen demonstrated to be high degree of sensitivity and specificity to ED [12-14]. Serkan Deveci et al. used the Doppler ultrasonography (DUS) to evaluate the ability of the IIEF differentiating different pathophysiologies of ED [15]. However, they concluded that the IIEF is not completely accurate in differentiating between organic and psychogenic ED. In addition, several studies [16-18] have confirmed that there is no significant difference in the IIEF or IIEF-5 scores among patients with specific vascular causes (i.e. arterial insufficiency, vascular leakage and mixed disorder).

However, few investigations about the differences in IIEF-5 among patients with all different pathophysiology have been reported. It is still unclear whether the IIEF-5 is capable of diagnosing the cause of ED. The present study aimed to evaluate the ability of the IIEF-5 to differentiate among all different pathophysiology of ED, according to standard comprehensive diagnostic procedures [8,19].

## Methods

We investigated consecutive patients with established ED in the urology or andrology clinics from three university affiliated hospitals (Xiangya Hospital, the Second Xiangya Hospital, and the Third Xiangya Hospital, Central South University) and two training hospitals (the First People’s Hospital of Chenzhou, the Central Hospital of Xiangtan) from January 2006 to January 2010. All subjects provided written informed consent. The project was organized by the Third Xiangya hospital, Central South University, and it was approved by the ethics committee of each institution. All protocols were reviewed and approved by the ethics committee of the Third Xiangya hospital, Central South University.

All patients (n = 3327) complained of the inability to achieve and/or maintain erection of sufficient rigidity and duration to permit satisfactory sexual performance. The patients completed the IIEF-5 at doctor’s office. Then they underwent independent evaluations by an experienced urologist or andrologist. The diagnostic steps and classification were based on EAU guidelines on erectile dysfunction (update March 2005) [8]. A detailed medical and sexual history was obtained and a physical examination was performed in all patients. Physical examination included the assessment of genitourinary, endocrine, vascular, and neurologic systems. A fasting glucose and lipid profile screen was performed if not assessed in the previous 12 months. Further laboratory testing included a morning sample of total testosterone and additional tests were determined when relevant cause was suspected. Patients with abnormal responses to examinations or tests were referred for specific tests (e.g. nocturnal penile tumescence and rigidity, NTPR).

The most primary cause was judged on the basis of the above-mentioned comprehensive diagnostic procedures. For example, they could be diagnosed as hormonal ED, when the patients with low sexual desire, abnormal physical examination (e.g. small testes, reduced body hair, gynecomastia), low testosterone level, or other related hormone abnormalities (e.g. LH, E_2,_ PRL, TSH, T3, T4). A patient will be diagnosed as psychogenic ED if no organic cause was detected, whereas he was suffered with mental disorders including performance anxiety, a strained relationship, psychological stress, lack of sexual arousability, and overt psychiatric disorders such as depression and schizophrenia (see Additional file 1).

176 subjects were excluded from this study due to the following reasons: (1) cause could not be determined by above procedures; (2) patients had two or more causes that are equally predominant; (3) patients were not willing to accept further investigations.

The severity of ED was evaluated using IIEF-5, and was classified into 4 levels based on the scores: severe (5–7), moderate (8–11), mild to moderate (12–16), and mild (17–21) [13].

### Statistical analysis

Qualitative variables were described by frequency, percentages, mean, and 95% confidence intervals. Nonparametric test was utilized: (1) Spearman correlations were used to assess the association between individual variables; (2) quantitative data of two individual samples were evaluated by Wilcoxon rank sum test; (3) multiple samples were evaluated by Kruskal-Wallis H test. Comparison between categorical variables was performed using chi-square test. Data analysis was performed using the SPSS 17.0 software (SPSS, Chicago, USA). The significance level was set at P = 0.05, and it was adjusted to 0.017 (0.05/3 = 0.017) for pairwise comparisons among the three vasculogenic subgroups (arteriogenic, venogenic, mixed vascular).

## Results

The median age of the enrolled subjects (n = 3,151) was 39 (interquartile range, 29.5-48.5) years, with a range from 19 to 70 years.

The primary causes of ED were psychogenic (1866 patients, 59.2%) and organic (1285 patients, 40.8%) respectively. The pathophysiological causes of organic ED were vasculogenic (52.1%, 670/1285), neurogenic (10.0%, 129/1285), anatomical/structural (6.9%, 89/1285), hormonal (17.4%, 224/1285), and drug-induced (13.5%, 173/1285), respectively.

**Figure 1:**
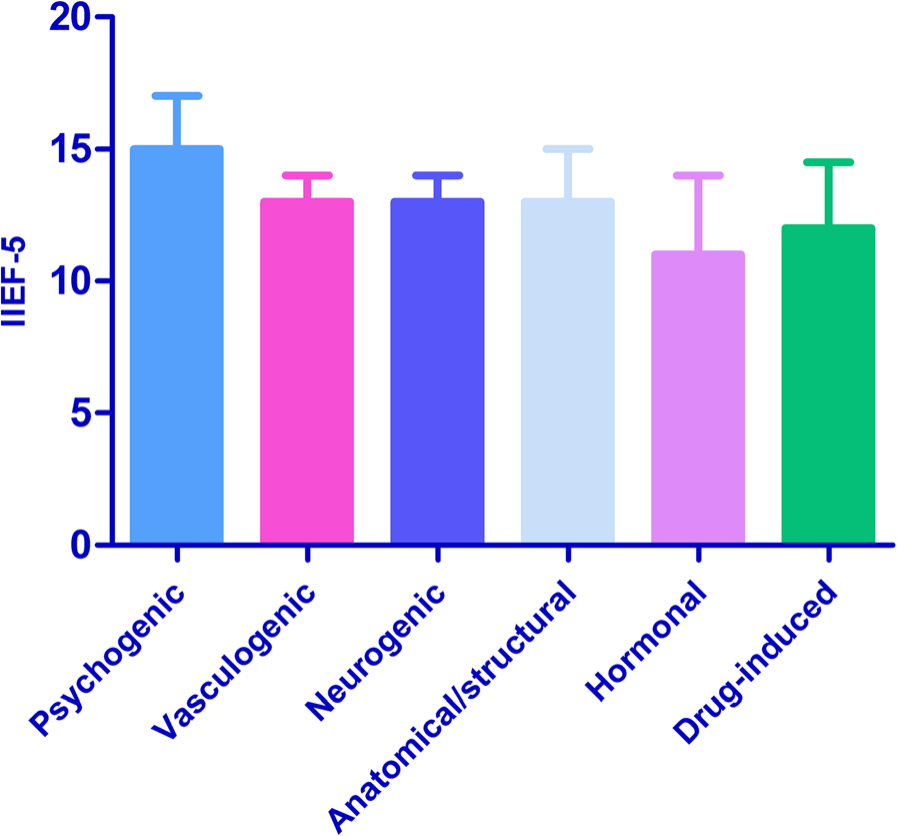
**The IIEF-5 scores of 3,151 patients with different pathophysiologic causes.** Error bar: interquartile range. Kruskal-Wallis H test for all groups, all organic groups (except for psychogenic): P = 0.000, and P = 0.073, respectively.

The IIEF-5 scores of patients with different primary causes were presented in Figure 1. The median IIEF-5 score of these subjects was 14 (interquartile range, 11.5, 16.5). A significant difference in the mean IIEF-5 score was observed between the subjects with psychogenic ED and those with organic ED (P = 0.000) (Table 1). Moreover, although there was a significant difference in the IIEF-5 scores among all groups (P = 0.000), no significant difference was identified in patients with organic causes (P = 0.073).

**Table 1:** Age distribution and median IIEF-5 scores of patients with different primary pathophysiologic causes

**Primary causes**	**<40 year**	**≥40 year**	**Overall**	**Median IIEF (IQR)**
Psychogenic	1145 (66.4)	721 (50.6)	1866 (59.2)	15 (13, 17)
Organic	580 (33.6)	705 (49.4)	1285 (40.8)	12 (9.5, 14.5)
Total	1725	1426	3151	14 (11.5, 16.5)

IQR, interquartile range. Data in parentheses are percentages of different age, unless otherwise noted.

Age: χ^2^ test for psychogenic versus organic: Pearson Chi-square = 80.858, P < 0.001. IIEF-5: Wilcoxon rank sum test for psychogenic versus organic: P < 0.001.

**Figure 2:**
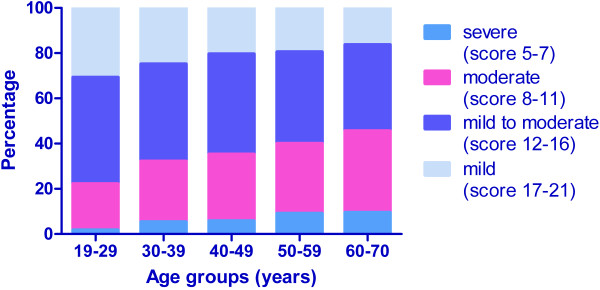
**Severity of ED increased with age.** Spearman correlations for 3151 patients: rs = −0.198, P = 0.000.

The distribution of ED severity in different pathophysiologic causes was presented in Table 2. Moreover, as indicated in Figure 2, the ED severity increased with age. A significant correlation was found between age and IIEF score.

**Table 2:** Patient severity and primary pathophysiologic cause erectile dysfunction

**Cause**	**Severe**	**Moderate**	**Mild to moderate**	**Mild**	**OR (95% CI)**	**P Value**
Psychogenic	17 (0.9)	356 (19.1)	859 (46.0)	634 (34.0)	1.00 (reference)	
Vasculogenic	83 (12.4)	225 (33.6)	281 (41.9)	81 (12.1)	1.29 (1.19-1.38)	0.000
Neurogenic	17 (13.2)	45 (34.9)	54 (41.9)	13 (10.1)	1.27 (1.17-1.39)	0.000
Anatomical/structural	8 (9.0)	26 (29.2)	42 (47.2)	13 (14.6)	1.25 (1.15-1.37)	0.000
Hormonal	17 (7.6)	111 (49.6)	81 (36.2)	15 (6.7)	1.23 (1.14-1.34)	0.000
Drug-induced	15 (8.7)	70 (40.5)	74 (42.8)	14 (8.1)	1.30 (1.19-1.41)	0.000
Overall	157 (5.0)	833 (26.4)	1391 (44.1)	770 (24.4)		

OR, odds ratio; CI, confidence interval. Data in parentheses are percentages, unless otherwise noted. Adjusted OR (for age) was measured by the ratio of sicker patients (severe and moderate, IIEF-5 scores of which are between 5 and 11, versus mild to moderate and mild, IIEF-5 scores of which are between 12 and 21 ) in every groups. The psychological ED group (IIEF-5 (5–11) / IIEF-5 (12–21)) was put as reference. CI, confidence interval.

The severity and age distribution of patients with various vasculogenic ED was shown in Table 3 and Table 4 , respectively. A significant difference was detected among the different vascular causes, the ED severity of patients with mixed vascular was higher than those with either arteriogenic or venogenic cause, but no difference was found between arteriogenic and venogenic causes (13 vs 13, P = 0.912 (adjusted α = 0.017)).

**Table 3:** Comparison of the severity of patients with different vascular causes according to IIEF-5

**Vascular cause n (%)**	**Severe**	**Moderate**	**Mild to moderate**	**Mild**	**Median IIEF (IQR)**
Arteriogenic	34 (12.9)	80 (30.4)	115 (43.7)	34 (12.9)	13 (10.5, 15.5)
Venogenic	34 (10.5)	106 (32.8)	141 (43.7)	42 (13.0)	13 (11–15)
Mixed vascular	15 (17.9)	39 (46.4)	25 (29.8)	5 (6.0)	11 (8.5-13.5)
Overall	83 (12.4)	225 (33.6)	254 (41.9)	85 (12.1)	13 (11, 15)

IQR, interquartile range. Data in parentheses are percentages, unless otherwise noted. Severity distribution: χ^2^ test for all groups, all groups (except for mixed vascular): Pearson Chi-square = 14.351, P = 0.026; Pearson Chi-square = 0.984, P = 0.805 (adjusted α = 0.017), respectively. IIEF-5: Kruskal-Wallis H test for all groups: P = 0.003. Wilcoxon rank sum test for arteriogenic versus venogenic: P = 0.912 (adjusted α = 0.017).

**Table 4:** Age and specific causes of vasculogenic ED

**Vascular cause n (%)**	**19-29**	**30-39**	**40-49**	**50-59**	**60-70**	**Median age (IQR)**
Arteriogenic	63 (24.0)	43 (16.3)	114 (43.3)	35 (13.3)	8 (3.0)	40 (32, 48)
Venogenic	81 (25.1)	94 (29.1)	108 (33.4)	34 (10.5)	6 (1.9)	39 (31.5-46.5)
Mixed vascular	11 (13.1)	22 (26.2)	32 (38.1)	16 (19.0)	3 (3.6)	41 (35–47)
Overall	155 (23.1)	159 (23.7)	254 (37.9)	85 (12.7)	17 (2.5)	40 (32.5, 47.5)

IQR, interquartile range. Data in parentheses are percentages, unless otherwise noted. Age distribution: χ^2^ test for all groups, all groups (except for mixed vascular): Pearson Chi-square = 30.416, P = 0.000; Pearson Chi-square = 19.24, P = 0.001 (adjusted α = 0.017), respectively. Age: Kruskal-Wallis H test for all groups: P = 0.003. Wilcoxon rank sum test for arteriogenic versus venogenic: P = 0.04 (adjusted α = 0.017).

Patients’ median duration of ED was 2 years (interquartile range, 0.5-3.5). There were significant differences in the duration among patients from each pathophysiology (Kruskal-Wallis H test for all groups, all groups (except for psychogenic), all organic groups (except for psychogenic and unknown): P = 0.000, P = 0.000, and P = 0.000, respectively). Meanwhile, the IIEF-5 score was negatively correlated with ED duration (rs = −0.189, P = 0.000, Spearman correlations). Furthermore, a significant difference in duration was detected among patients with three vascular causes (P = 0.046), but no significant difference in duration was found among arbitrary vascular causes (arteriogenic versus venogenic, arteriogenic versus mixed, venogenic versus mixed, P = 0.538, 0.019, and 0.027 respectively (adjusted α = 0.017).

## Discussion

ED is a serious and growing public health problem. The prevalence of ED increased with age [20]. In most countries, a large proportion of patients visiting outpatient clinics were older than 40 years [21,22]. However, our results indicated that the number of young (<40 years) ED patients were more than ones over 40 years (Table 1). Similar results were also obtained in several studies of Chinese ED patients [23,24]. In a 5-year survey in 11 cities in China, Liu et al. found that most patients were younger than 50 years old (75.7% in 2003 and 74.5% in 2008) [23]. Another study in older (>40 years) patients in Beijing also showed that only 27.4% of them recognized ED as a disease, and just 12.1% visited their doctors [24]. These were likely associated with more conservative culture in China [10,25].

ED has serious effects on men’s physical and mental health [26,27]. According to different pathogenic mechanisms, ED has usually been classified as psychogenic, organic (ie, neurogenic, hormonal, vasculogenic, anatomic/structural, or drug-induced), or mixed psychogenic and organic causes [8,28]. If ED is longstanding, this may build patient’s own world of fear, anxiety, worry, depression and distress around his disorder [29,30]. Hence, the mixed form is generally regarded as the most common category in most studies [2,31]. In this study, we also found that nearly half of subjects have combined different causes. Their combined causes are not necessarily the same with each other. Hence, it is not conducive to analyze the ED causes if this large number of patients all just classified as “the mixed form”. Therefore, the primary causes of subjects were studied in this investigation to avoid confusion in patients with mixed causes. The primary cause was determined by an experienced urologist or andrologist according to the comprehensive diagnostic procedures (for details see Methods and Additional file 1). It has been confirmed that the comprehensive procedures are superior in establishing the causes of ED [19,32]. Under some circumstances doctors could not draw a clear distinction between the primary cause and the minor cause. These cases (176/3327, 5.3%) were excluded from present investigation. This proportion was similar to a study by Hatzichristou et al. (78/1276, 6.1%) [19].

IIEF-5 is a brief, validated, multidimensional, self-administered tool to assess erectile function [13,33]. Rosen RC, et al. believed that there are still many potential areas for future research on the IIEF [34]. For example, the IIEF may not be completely accurate in differentiating among various sub-groups [34,35].

The aim of this study was to assess the difference among all the different pathophysiologies of ED in IIEF-5 by analysing a multicenter, large sample of ED patients using the comprehensive procedures. Previous studies have compared the IIEF or IIEF-5 between organic and psychogenic ED, or between arteriogenic and cavernosal ED. Serkan Deveci et al. compared the IIEF scores with the results of flow dynamics analysis, using the penile duplex Doppler ultrasonography (DUS) in 112 patients. They found that the IIEF was not completely accurate in differentiating between organic and psychogenic ED, but have potential ramifications for evaluating the baseline severity of ED in trials of erectogenic agents. All patients with venogenic cause (4/4) were all categorized as severe ED according to IIEF score, and none of the patients (0/16) with venous leak (including venogenic cause and mixed vascular cause) had mild ED. However, more than a fifth (6/28) of the men with normal erectile haemodynamics also were classified as severe ED [15]. Melman A, et al. used the Rigiscan results to assess the ability of the IIEF-6 in 32 consecutive patients. Similarly, no significant correlation was found between NPTR data and IIEF-6 score. They concluded that the IIEF-6 is unable to differentiate between various causes of ED [35].

In addition, several studies [16-18] also suggestted that there is no significant difference in the IIEF or IIEF-5 scores among patients with specific vascular causes (i.e. arterial insufficiency, vascular leakage and mixed disorder). Our data indicated that not only there is no significant difference between arteriogenic and venogenic cause in IIEF-5, but also these causes have a similar severity distribution (Table 3). Hence, all these results conducted that IIEF-5 is not accurate in distinguishing the specific causes of vasculogenic ED.Further, in the current study, although we found a statistical difference among all different pathophysiologies in IIEF-5 (P = 0.000), no significant difference were found among all kinds of organic causes (P = 0.067 or P = 0.073 (except for unknown)) (Figure 1). Hence, the main difference in IIEF-5 was between psychogenic and all other causes. So the IIEF-5 did not statistically differentiate the specific organic causes of ED as determined by evidence-based testing by the comprehensive diagnostic procedures, but it had potential ramifications for the evaluation of whether the patients were psychological origin.

Our study has several limitations. Firstly, it is not population based. However, the patients seeking treatment at clinics were not chosen on purpose. Rather, they were consecutive patients who were diagnosed with erectile dysfunction by urologist/andrologist. Although these results could not represent the epidemiological characteristics in ED population, it could be more useful for clinical urologists/andrologists. Secondly, all information was recorded by the physicians, thus, self-reported questionnaire (except for IIEF-5) for patients is lacking. This could have resulted in information loss, particularly regarding the demographic characteristics. Thirdly, the contribution of the relational factors (e.g. the types of drugs that can induce ED) in patients were less evaluated, which should be enhanced in follow-up. Finally, an objective, quantitative criteria for judging primary pathophysiology are lacking. Hence, the identification of primary pathophysiological cause depends in part on the physician’s judgment, which means inevitable subjectivity. However, this subjective bias could be reduced by basing the identification on uniform guidelines in the diagnostic procedures. We also plan to use statistical methods (e.g. discriminant analysis) to narrow this bias in further research.

## Conclusions

This multicenter study was conducted in a large sample of ED patients seeking treatment at clinics. It indicated that the age of Chinese outpatients with ED was younger than other countries. Moreover, no significant difference in IIEF-5 scores was observed among ED patients with different organic pathophysiologies. Therefore, IIEF-5 was not completely sufficient to distinguish the ED pathophysiology, and it should be used with caution.

## Abbreviations

IIEF-5: The simplified International Index of Erectile Function; ED: Erectile dysfunction; IQR: Interquartile range; PDE5-i: Phosphodiesterase type 5 inhibitors; DUS: Doppler ultrasonography; NTPR: Nocturnal penile tumescence and rigidity; OR: Odds ratio; CI: Confidence interval.

## Competing interests

All the authors declare that they have no financial competing interests.

## Authors’ contributions

YT participated in the conception and design of entire study; ZT performed the data acquisition, and helped to draft the manuscript;,DL performed the statistical analysis, and coordination and helped to draft the manuscript; XbZ helped to draft the manuscript. LY, performed the data acquisition; XsZ performed the data acquisition; XyZ performed the data acquisition; All authors read and approved the final manuscript.

## Pre-publication history

The pre-publication history for this paper can be accessed here:

http://www.biomedcentral.com/1471-2490/14/52/prepub

## Supplementary Material

Additional file 1 **Self-developed diagnostic criteria for primary pathophysiological cause of ED (based on EAU guidelines on erectile dysfunction **^**8**^**).** Click here for file
