# Supplementary material for: Comparison of the simplified International Index of Erectile Function (IIEF-5) in patients of erectile dysfunction with different pathophysiologies
Source: BMC Urol. 2014 Jul 5;14:52. doi: 10.1186/1471-2490-14-52 (PMC4094415; doi:10.1186/1471-2490-14-52)
Supplement: Additional file 1 — Self-developed diagnostic criteria for primary pathophysiological cause of ED (based on EAU guidelines on erectile dysfunction 8). [file 1471-2490-14-52-S1.doc]

**Additional file 1:** Self-developed diagnostic criteria for primary pathophysiological cause of ED （based on EAU guidelines on erectile dysfunction ^8^）

| pathophysiological cause | Clinical manifestation | History | Laboratory and specific tests |
| --- | --- | --- | --- |
| Psychogenic | Decreased libido, physical examination is normal. | Performance anxiety,relationship problems, psychological stress, depression, schizophrenia, postmyocardialinfarction etc.. | Normal NTPR, ICI Duplex ultrasound, and BCR. |
| Vasculogenic | Normal libido, physical examination is abnormal in vascular system. | Cardiovascular disease, hypertension, diabetes, hypercholesterolemia, smoking, major surgery or radiotherapy etc.. | Abnormal NTPR and Duplex ultrasound. |
| Neurogenic | Neurological examination is abnormal. | Multiple sclerosis, multiple atrophy, stroke, Alzheimer’s disease, Parkinson’s disease, tumours, spinal cord disorders, alcoholism, uraemia, polyneuropathy, diabetic neuropathy, surgery, radiotherapy, pelvic trauma, etc.. | Abnormal BCR and NTPR. Normal ICI. |
| Anatomical/structural | Abnormal penis. | Peyronie’s disease, penile fracture, congenital curvature of the penis, micropenis, hypospadias, epispadias and etc.. | N/A. |
| Hormonal | Decreased libido and nocturnal erections. Genitourinary or endocrine examination is abnormal. | Hypogonadism, hyperprolactinemia, hyper- and hypothyroidism, Cushing’s disease, tumor, injury or surgery to the testis or brain etc.. | Abnormal NTPR and hormone (e.g. TT, LH, E_2_, PRL, TSH, T3, T4) level, and normal ICI. |
| Drug-induced | N/A. | History of relevant drug, i.e. antihypertensives, antidepressants, antipsychotics, antiandrogens, antihistamines, recreational drugs, etc.. | Abormal NTPR and Duplex ultrasound. |

**Reference values:**

Total testosterone: (TT): ≥3.5ng/mL; luteinizing hormone (LH): 1.70~8.60 mUI/mL; estradiol (E_2_): 49.6~218 pmol/L; Prolactin (PRL): 4.13~18.40 ng/ml; Thyroid Stimulating Hormone (TSH): 0.35~4.94μU/mL; Triiodothyronine (T3): 3.54~6.5pmol/L; thyroxine (T_4_): 10.68~22.7pmol/L

Nocturnal penile tumescence and rigidity (NTPR): NEVA: more than 3 events, duration of events>15min, percentage of volume change over baseline>200%; Rigiscan: more than 3 events, duration of events>10min, erectile rigidity>60%.

ICI: A normal response is a rigid erectile response (unable to bend the penis) that appears within 10 min after the intracavernous injection and lasts for 30 min. Duplex ultrasound: PSV>30cm/s, EDV<5cm/s, and RI>0.8. Bulbocavernosus reflex (BCR): 27~42ms.
